# Preoperative Nutritional Status and Clinical Complications in the Postoperative Period of Cardiac Surgeries

**DOI:** 10.5935/1678-9741.20160077

**Published:** 2016

**Authors:** Luciana de Brito Gonçalves, Natanael Moura Teixeira de Jesus, Maiara de Brito Gonçalves, Lidiane Cristina Gomes Dias, Tereza Cristina Bomfim de Jesus Deiró

**Affiliations:** 1 Universidade Federal da Bahia (UFBA), Salvador, BA, Brazil.

**Keywords:** Nutrition Assessment, Postoperative Complications, Cardiac Surgical Procedures, Nutritional Status

## Abstract

**Objective:**

This study aims to assess the preoperative nutritional status of patients and the role it plays in the occurrence of clinical complications in the postoperative period of major elective cardiac surgeries.

**Methods:**

Cross-sectional study comprising 72 patients aged 20 years or older, who underwent elective cardiac surgery. The preoperative nutritional assessment consisted of nutritional screening, anthropometry (including the measurement of the adductor pollicis muscle thickness) and biochemical tests. The patients were monitored for up to 10 days after the surgery in order to control the occurrence of postoperative complications. The R software, version 3.0.2, was used to statistically analyze the data.

**Results:**

Clinical complications were found in 62.5% (n=42) of the studied samples and complications of non-infectious nature were most often found. Serum albumin appeared to be associated with renal complications (*P*=0.026) in the nutritional status indicators analyzed herein. The adductor pollicis muscle thickness was associated with infectious complications and presented mean of 9.39±2.32 mm in the non-dominant hand (*P*=0.030). No significant correlation was found between the other indicators and the clinical complications.

**Conclusion:**

The adductor pollicis muscle thickness and the serum albumin seemed be associated with clinical complications in the postoperative period of cardiac surgeries.

**Table:** 

Abbreviations, acronyms & symbols	
**AIDS**	**= Acquired immunodeficiency syndrome**
**APM**	**= Adductor pollicis muscle**
**BMI**	**= Body mass index**
**CCU**	**= Coronary care unit**
**CPB**	**= Cardiopulmonary bypass**
**CVDs**	**= Cardiovascular diseases**
**HED**	**= Hydroelectrolytic disorders**
**NRS-2002**	**= Nutritional risk screening**
**TLC**	**= Total lymphocyte count**

## INTRODUCTION

According to the World Health Organization (2011), approximately 17 million people have died due to cardiovascular diseases (CVDs), *i.e.,* three in each ten deaths^[1]^. Among these 17 million, 7 million died of ischemic heart disease and 6.2 million died due to cerebrovascular accident^[1]^.

Once the risk factors worsen or trigger the development of CVD, it is necessary to perform cardiac surgeries^[2]^. Myocardial revascularization and valve replacement stand out among these surgeries^[2,3]^. According to the Ministry of Health, approximately 275,838 circulatory system surgeries^[4]^ were performed in 2013.

Cardiac surgeries lead to metabolic changes and they may be defined as complex procedures responsible for relevant organic repercussions associated with changes in the physiological mechanisms^[5]^. Thus, several studies have shown great interest in studying clinical complications in the postoperative period of cardiac surgeries^[6-10]^. The time of hospital stay is also a factor of great relevance. A study found that the mean hospital stay time after cardiac surgeries is of approximately 10 days in the Brazilian Northeastern region; however, no time variation was found between different regions^[3]^.

Therefore, assessing the preoperative nutritional status may help adopting early nutritional interventions to patients at high risk of developing postoperative complications^[5]^. Thus, the preoperative nutritional status should be an important indicator to the selection of patients supposed to undergo surgery^[11]^. In light of the foregoing, the aim of the current study is to investigate the association between nutritional status and clinical complications in the postoperative period of major elective cardiac surgeries.

## METHODS

The present study followed a cross-sectional design and it was conducted at Ana Nery Hospital and at Professor Edgard Santos Hospital Complex (COM-HUPES Complexo Hospitalar Professor Edgard Santos). Both hospitals are located in Salvador, Bahia State, Brazil.

The sample size was estimated through the 58% prevalence of postoperative clinical complications found in the literature^[10]^. A standard deviation of 12 was adopted. A significance level of 5% was adopted to reject the equality hypothesis between ratios. The sample size was increased by 10%, due to the possibility of losses and refusals, thus totaling 72 individuals.

Individuals from both genders, in the age group 20 years or older, who had undergone major elective cardiac surgery between August 2013 and January 2014, were included in the study. The following patients were not included in the study: patients subjected to angioplasty, pacemaker implantation and emergency cardiac surgery; patients who showed medical and physical conditions that prevented weighing or anthropometric measurements; patients diagnosed with infection, hepatic or renal dysfunctions; pregnant women; patients with acquired immunodeficiency syndrome (AIDS), cancer, and severe obesity (body mass index ≥ 40 kg/m^2^); patients who refused to continue in the study; and patients with medical records missing relevant data. The preoperative variables were collected in the patients' records, namely: gender, age, clinical diagnosis, left ventricular ejection fraction and smoking history.

The study protocol was approved by the Ethics Committee of the COM-HUPES- Federal University of Bahia, Brazil (385.042/2013). All subjects were informed about the aim of the present study, both orally and written. A written informed consent document was signed by the participants. The informed consent was in compliance with Resolution 466/12 of the National Health Council and Declaration of Helsinki.

### Variables Analyzed in the Preoperative Period

The patients underwent nutritional assessment after the surgical indication was confirmed. The following parameters were used: 1. Nutritional screening - performed within 72 hours after hospitalization; 2. Anthropometry; 3. Preoperative Biochemical Testing: serum albumin, lymph cytometry, total cholesterol, LDL-c (Low Density Lipoproteins), HDL-c (High Density Lipoproteins), and triglycerides. Data of all examinations were collected from the medical records of the patients.

The nutritional screening of the patients was performed through the NRS2002 (Nutritional Risk Screening) "score", according to disease severity (classified as mild, moderate and severe), to weight loss in the last three months, to food intake, and to body mass index (BMI)^[11]^. After the summation, the participants were classified as "no nutritional risk" (score lower than three) and "at nutritional risk" (score higher than or equal to three)^[11]^.

The anthropometric assessment consisted of measurements such as: usual weight, percentage of unintentional weight loss in the last six months, current weight, BMI calculation (kg/m^2^), arm circumference, triceps skinfold, waist circumference, adductor pollicis muscle (APM) thickness in the dominant (right) and non-dominant hand, subscapular skinfold, sum of the two folds (triceps and subscapular), height, and corrected arm muscle area. The knee height was used to estimate height. Arm muscle and calf circumferences were used to assess the muscle mass of patients age 60 years or older. The anthropometric parameters obtained herein were used according to the previously described methods^[11-14]^, which were interpreted through percentile-reference tables by taking age and gender into consideration^[15]^.

The weight loss percentage was used to calculate the difference between usual weight and current weight divided by the usual weight. Values higher than 10% were classified as significant or severe weight loss^[16]^. The patients were weighed on the Glass 200 G Tech^®^ digital scale, with 0.1 g accuracy and maximum capacity 200 kg. Height was measured in the Seca^®^ portable stadiometer with 2.20-m scale. The skinfolds^[13]^ and the APM^[14]^ were measured using the Lange^®^ scientific skinfold caliper. The circumferences were measured using the TBW^®^ inelastic tape. The height of the elder patients was estimated according to leg length (knee height) using the Caumaq^®^ infantometer.

The BMI resulted from the division of the current weight (kg) by the squared height (m). The BMI-based nutritional status classification followed the criteria suggested by World Health Organization (2000) and Lipschitiz (1994) for adults and seniors, respectively^[17,18]^.

The waist circumference was measured two centimeters above the umbilicus in order to standardize the measures and it was classified according to the cut-off points established herein^[17]^. The APM was measured according to the described technique^[14]^. All the herein described anthropometric measurements were performed in duplicate, and the mean of each measure was considered as real value. The three appraisers have been properly trained to minimize interappraiser errors. The calculation of the total lymphocyte count (TLC) was performed as described in the literature^[16]^.

### Variables Analyzed in the Postoperative Period

The transoperative variables recorded herein were: heart surgery type, cardiopulmonary bypass (CPB) and anoxia times (minutes); time (days) on mechanical ventilation; time (days) in the coronary care unit (CCU), hospitalization time (days), and death events.

Surgical complications were assessed for up to 10 days after surgery, according to the time estimated in studies about hospital stay in the postoperative period of cardiac surgeries^[3]^. The complications were stratified as: Cardiac complications - acute myocardial infarction, low cardiac output syndrome, and atrial fibrillation; Pulmonary complications - tracheal intubation for more than 48 hours after surgery, atelectasis, bronchoconstriction, acute respiratory distress syndrome, acute respiratory failure, pleural effusion, mechanical ventilation-associated pneumonia, and pneumothorax; Renal complications - increased serum creatinine equal to 0.3 mg/dL, decreased urine output, and need of dialysis at any time after surgery; Infectious complications - lung, urinary tract and surgical site infections, mediastinitis, and endocarditis; Gastrointestinal complications - mesenteric ischemia, gastrointestinal bleeding, and abdominal inflammation or obstruction; Neurological complications - cerebrovascular accident; Hematologic complications - bleeding, thrombotic events and hydroelectrolytic disorders (HED)^[10]^.

### Statistical Analysis

The database was developed in Excel 2010 and analyzed in the R software (version 3.0.2). The descriptive analysis (absolute/relative frequency, mean, standard deviation, and median) was used to identify the general and specific features of the studied sample. The Chi-square test or the Fisher's exact test was used to check the associations between the qualitative variables and the occurrence of complications. The Student's t test or the non-parametric Mann-Whitney test was used to find the associations between the quantitative variables and the occurrence of complications. The significance level was set at *P*<0.05. The results obtained herein are presented in the tables and charts developed in Word 2010.

## RESULTS

The studied population comprised 72 male and female patients at mean age 52.2±14.5 years; 41.6% (n=30) of these patients were elder, and 50% (n=36) of them were women. According to the nutritional screening, only 8.3% (n=6) of the patients were at nutritional risk. Sample featuring is described in Table 1.

**Table 1:** Characterization of patients subjected to major elective cardiac surgery.

Variables	n=72	%
**Age**		
≥ 60 years	30	41.6
**Gender**		
Female	36	50
**Smoking**		
Abstainer	36	50
**Ethnic group**		
Non-white	67	93.1
**Previous heart surgery**	12	16.7
**NRS-2002**		
At nutritional risk	6	8.3
**Body mass index classification**		
Underweight	10	13.9
Overweight	37	51.4
**Clinical diagnosis**		
Coronary artery disease	28	37.5
Congestive heart failure	23	31.9
Rheumatic heart disease	23	31.9
Mitral failure	18	25
Aortic insufficiency	10	13.9
Aortic stenosis	10	13.9
Atrial fibrillation	7	9.7
Acute myocardial infarction	5	6.9
Pulmonary hypertension	4	5.6
Congenital heart disease	2	2.8
Chagas cardiomyopathy	2	2.8

It was observed that 51.4% (n=37) of the patients subjected to cardiac surgery were classified as overweight; however, 13.9% (n=10) of them were underweight (Table 1). The anthropometric profile was featured through mean BMI 26.2±4.3 kg/m^2^ (Table 2), minimum 18.4 kg/m^2^ and maximum 36.5 kg/m^2^.

**Table 2:** Clinical, anthropometric, biochemical and postoperative features of patients undergoing surgical procedure.

Variables	Mean	Median	Standard deviation
Age (years)	52.2	52.5	14.5
Ejection fraction (%)	62.3	62	10.4
Body mass index (kg/m^2^)	26.2	26.4	4.3
Waist circumference (cm)	91.9	93.2	11.6
Corrected arm muscle area (cm^2^)	37.9	37.5	12.4
Arm muscle circumference (cm)	23.7	24.3	3.5
Calf circumference (cm)	35.2	34	4
Dominant adductor pollicis muscle (mm)	11.4	11	3.4
Non-dominant adductor pollicis muscle (mm)	11.2	10.4	3.5
Triceps skinfold (mm)	16.5	15.3	5.3
Sum of the two skinfolds (mm)	37.8	37.3	12.2
Albumin (g/dL)	3.8	3.8	0.4
Total lymphocyte count (mm^3^)	2.128	2.135	799.9
Total cholesterol (mg/dL)	185.6	172.5	59.8
HDL-c (mg/dL)	38.5	38	11.4
LDL-c (mg/dL)	115.5	112.5	47
Triglycerides (mg/dL)	159	144.0	132.8
Length of anoxia (min)	74.2	65	40.5
Extracorporeal circulation (min)	91.8	80	43.9
Mechanical ventilation (days)	1.5	1	1.8
Stay in the coronary care unit (days)	4	3	4.5
Length of hospital stay (days)	29.3	21.5	20.5

HDL-c-=high-density lipoprotein cholesterol; LDL-c=low density lipoprotein cholesterol

The analysis of body compartment distribution showed significant distribution of adipose tissue excess in adults, 66.7% (n=28); however, 11.9% (n=5) of the patients showed depleted adipose tissue. The highest rate of elderly patients showed adequate fat reserve, 83.3% (n=25). The present study shows that 14.3% (n=6) of the adult individuals presented depleted muscle reserves; however, the highest rate of it was found among the elderly patients, 33.3% (n=10). The mean values of these measures and of other nutritional status parameters are shown in Table 2. The mean APM thickness in the dominant hand was 11.4±3.4 mm, and that in the non-dominant hand was 11.2±3.5 mm (Table 2).

According to the biochemical analysis, 12.5% (n=9) of the patients showed hypoalbuminemia and 38.9% (n=28) of them had some degree of depletion, which was demonstrated through the TLC. Table 2 describes these findings.

Transoperative variables such as mean CPB and mechanical ventilation time are shown in Table 2. Only one of the patients included in the study was not subjected to CPB. The mean CCU stay time was 4±4.5 days, it ranged from 1 to 34 days, and the mean hospital stay time was 29.3±20.5 days (Table 2), minimum 9 and maximum 118 days.

**Fig. 1:**
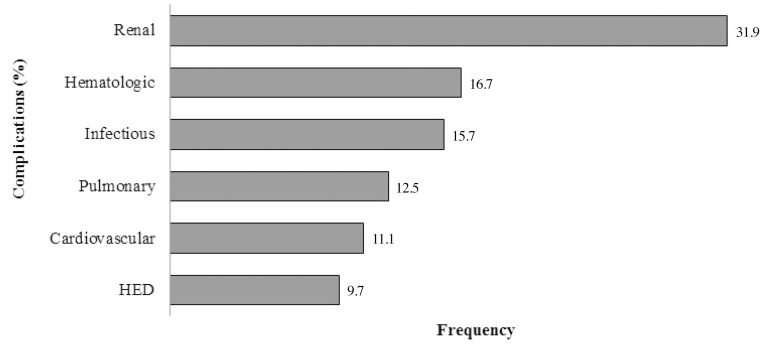
Distribution of clinical complications found in the postoperative period of major elective cardiac surgeries. HED=hydroelectrolytic disorders

Postoperative clinical complications affected 62.5% (n=45) of the studied patients, and non-infectious complications were the most common ones (Figure 1); however, no patient presented gastrointestinal and neurological complications. As for the observed postoperative complications, 38.9% (n=28) of the patients presented at least one type of complication. Some of them had up to four different types of complications.

Age was not associated with postoperative complications (*P*=0.077). In addition, no significant association with gender was found in NRS 2002 (Table 3).

**Table 3:** Association between anthropometric and transoperative variables and the occurrence of postoperative complications in individuals undergoing major elective cardiac surgeries.

Variables	Complication *		*P*-value
	Absent Mean/SD	Present Mean/SD	
Age (years)	48.3±12	54.2±15.5	0.077^[1]^
Dominant APM (mm)	12.3±4	10.7±2.9	0.217^[2]^
Non-dominant APM (mm)	12.1±4.2	10.5±2.9	0.148^[2]^
Extracorporeal circulation (min)	74.6±34.1	102.3±46.3	0.007^[2]^
Length of stay in CCU (days)	2.4±0.8	5.0±5.5	0.001^[2]^
Mechanical ventilation (days)	1.5±0.4	1.6±2.2	0.615^[2]^
**Variables**	**n (%)**	**n (%)**	*P*-value
**Gender**			0.465^[3]^
Female	12 (33)	24 (67)	
Male	15 (42)	21 (58)	
**Weight loss**			1.000^[4]^
No loss	25 (38)	41 (62)	
Significant loss	----	1 (100)	
Severe loss	2 (40)	3 (60)	
**NRS-2002**			0.400^[4]^
No nutritional risk	26 (39)	40 (61)	
At nutritional risk	1 (17)	5 (83)	
**Body mass index**			0.252^[3]^
Underweight	2 (20)	8 (80)	
Eutrophia	8 (32)	17 (68)	
Overweight	17 (46)	20 (54)	
**Triceps skinfold**			0.565^[4]^
Depletion of adipose tissue	2 (40)	3 (60)	
Good fat reserve	5 (20)	20 (80)	
**Summation of the two skinfolds**			0.186^[4]^
Depletion of adipose tissue	1 (20)	4 (80)	
Good fat reserve	3 (33)	6 (67)	
Excess adipose tissue	16 (57)	12(43)	
**Waist circumference**			0.350^[4]^
Normality	7 (32)	15 (68)	
Increased risk	7 (30)	16 (70)	
Very increased risk	13 (48)	14 (52)	
**Calf circumference**			0.431^[4]^
< 31 cm	1 (50)	1 (50)	
> 31 cm	6 (22)	21 (78)	
**Corrected arm muscle area**			0.665^[4]^
Underweight	2 (33)	4 (67)	
Good muscle mass reserves	18 (50)	18 (50)	
**Arm muscle circumference**			0.657^[4]^
Muscle mass depletion	3 (30)	7 (70)	
Good muscle mass reserves	4 (20)	16 (80)	
**Albumin**			1.000^[4]^
< 3.5 g/dL	3 (33)	6 (67)	
≥ 3.5 g/dL	24 (38)	39 (62)	
**Total lymphocyte count**			0.807^[4]^
> 1.500 cel/mm^3^	16 (36)	28 (64)	
1.500 – 1.200 cel/mm^3^	7 (35)	13 (65)	
800 – 1.200 cel/mm^3^	2 (67)	1 (33)	
< 800 cel/mm^3^	2 (40)	3 (60)	
**Type of surgery**			
Myocardial revascularization	13 (39)	20 (61)	0.760^[3]^
Mitral valve replacement	6 (25)	18 (75)	0.121^[3]^
Tricuspid valve surgery	2 (33)	4 (67)	1.000^[4]^
Mitral valve surgery	5 (62)	3 (38)	0.142^[3]^
Aortic valve replacement	6 (26)	17 (74)	0.171^[3]^
Correction of congenital anomalies	---	1 (100)	1.000^[4]^

* Presentation of at least one clinical complication in the postoperative period. SD=Standard deviation; APM=adductor pollicis muscle; CCU=length of stay in the coronary care unit

[1] Descriptive level of probability by the Student’s t-test.

[2] Descriptive level of probability by the nonparametric Mann-Whitney U-test.

[3] Descriptive level of probability by the chi-square test.

[4] Descriptive level of probability by the Fisher’s exact test.

Although a large number of overweight patients have shown complications, there was no significant association between the BMI categories and the occurrence of postoperative complications (Table 3). The same happened with the serum albumin levels; approximately 66.7% (n=6) of the patients who had hypoalbuminemia showed postoperative clinical complications; however, they had no statistical significance. The TLC was another laboratory parameter used in the current study, and it showed no association with the occurrence of complications. However, according to the aforementioned indicator, 60.7% (n=17) of the individuals who experienced complications had some degree of depletion (Table 3). The mean APM thickness showed no significant difference (*P*=0.217) in both hands, as shown in Table 3.

The CPB and CCU times were significantly higher in patients with postoperative complications (*P*=0.007 and *P*=0.001, respectively). The mean time in mechanical ventilation was slightly higher in individuals who had complications. However, there was no significant relationship with the occurrence of clinical complications in the postoperative period of cardiac surgeries (Table 3).

Table 4 shows the stratification of postoperative clinical complications. It was observed that 66.7% (n=6) of the individuals who had hypoalbuminemia presented renal complications (*P*=0.026). However, the other nutritional status variables were not associated with postoperative clinical complications.

**Table 4:** Association between the presence of clinical complications in the postoperative period of cardiac surgery and the nutritional status variables.

Variables	Complication*											
	Cardiac		Pulmonary		Renal		Infectious		Hematologic		HED	
	n (%)	*P*-value	n (%)	*P*-value	n (%)	*P*-value	n (%)	*P*-value	n (%)	*P*-value	n (%)	*P*-value
**BMI**		0.454^[1]^		0.186^[1]^		0.262^[2]^		0.821^[1]^		0.829^[1]^		1.000^[1]^
Underweight	2(20)		3 (30)		5 (50)		2 (20)		1 (10)		1 (10)	
Eutrophia	3 (12)		2 (8)		9 (36)		3 (12)		5 (20)		2 (8)	
Overweight	3 (8)		4 (11)		9 (24)		6 (16)		6 (16)		4 (11)	
**TSF**		1.000^[1]^		1.0^[1]^		0.336^[1]^		0.556^[1]^		0.538		1.000^[1]^
Depletion	1 (20)		1 (20)		1 (20)		0		1 (20)		0	
Eutrophia	4 (16)		4 (16)		13 (52)		5 (100)		3 (12)		4 (16)	
**2D summation**		1.000^[1]^		0.554^[1]^		0.540^[1]^		1.000^[1]^		1.000^[1]^		0.704^[1]^
Depletion	0		1 (20)		2 (40)		1 (20)		1 (20)		0	
Eutrophia	1 (11)		1 (11)		2 (22)		1 (11)		2 (22)		0	
Excess	2 (7)		2 (7)		5 (18)		4 (14)		5 (18)		3 (11)	
**Albumin**		1.000^[1]^		0.590^[1]^		0.026^[1]^		0.337^[1]^		0.340^[1]^		0.585^[1]^
< 3.5 g/dL	1 (11)		0		6 (67)		0		0		0	
≥ 3.5 g/dL	7 (11)		9 (14)		17 (27)		11 (17)		12 (19)		7 (11)	
**TLC**		0.888^[1]^		0.668^[1]^		0.233^[1]^		0.743^[1]^		0.564^[1]^		0.154^[1]^
> 1.500	5 (11)		5 (11)		13 (29)		8 (18)		6 (14)		3 (7)	
1.500 – 1.200	3 (15)		4 (20)		9 (45)		2 (10)		5 (25)		2 (10)	
800 – 1.200	0		0		1 (33)		0		0		0	
< 800	0		0		0		1 (20)		1 (20)		2 (40)	

* Presented some of the specified complications.

HED=hydroelectrolytic disorders; BMI=body mass index; TSF=triceps skinfold; 2D summation=sum of the two skinfolds; TLC=Total lymphocyte count

[1] Descriptive level of probability by the Fisher’s exact test.

[2] Descriptive level of probability by the chi-square test.

The CPB and CCU times showed statistically significant association with the occurrence of cardiac complications, as shown in Table 5. The time in mechanical ventilation and the CCU time were strongly associated with pulmonary complications. Age, CPB time and CCU time were associated with renal complications. Only the thickness of the non-dominant APM was associated with infectious complications (Table 5).

**Table 5:** Association between the pre-and postoperative variables and the presence of postoperative complications in patients undergoing cardiac surgery.

	Complication*									
Variables	Cardiac		Pulmonary		Renal		Infectious		Hematologic	
	Mean/SD	*P*-value	Mean/SD	*P*-value	Mean/SD	*P*-value	Mean/SD	*P*-value	Mean/SD	*P*-value
Age (years)^[1]^	59.5±15.1	0.134	55.9±16.9	0.421	57.9±15.1	0.023	54.0±17.7	0.660	45.2±16.8	0.066
EEC (min)^[2]^	142.1±67.8	0.020	124.6±54.8	0.054	116.0±53.8	0.005	102.3±48.4	0.499	102.5±44.8	0.282
DAPM (mm)^[2]^	11.9±5.2	0.534	11.1±2.6	0.401	11.5±3.2	0.889	9.8±2.5	0.157	11.1±1.5	0.988
NDAPM (mm)^[2]^	11.6±5.2	0.732	11.0±2.4	0.590	11.3±3.4	0.981	9.4±2.3	0.030	10.8±1.6	0.844
MV (days)^[2]^	2.9±4.9	0.456	3.6±4.5	0.001	2.3±3.1	0.083	1.8±1.5	0.232	1.3±0.9	0.435
CCU (days)^[2]^	6.8±4.7	0.006	10.4±9.9	0.001	7.0±7.0	0.000	7.2±9.4	0.095	6.0±9.1	0.641

* Presented some of the specified complications.

SD=standard deviation; ECC=extracorporeal circulation; DAPM=adductor pollicis muscle in the dominant hand; MAPND=adductor pollicis muscle in the non-dominant hand; MV=mechanical ventilation; CCU=length of stay in the coronary care unit

[1] Descriptive level of probability by the Student’s t-test.

[2] Descriptive level of probability by the nonparametric Mann-Whitney U-test.

Five point five percent (5.5%) (n=4) of the patients died. However, the postoperative mortality was not associated with any of the analyzed variables.

## DISCUSSION

The nutritional risk before the cardiac surgery has been associated with adverse effects in the postoperative period^[5,19]^ due to increased catabolism and metabolic requirements^[5]^.

The NRS 2002 is a nutritional screening instrument used to identify nutritional risk in hospitalized patients^[11]^. A small portion of the herein studied population was classified as "at nutritional risk", and these data were lower than those found by other authors^[11,19]^.

Lomivorotov et al.^[19]^ assessed the predictive value from nutritional status screening instruments applied to patients undergoing cardiac surgery. They found that the NRS 2002 method was the least sensitive to malnutrition detection. Such finding supports the importance of developing an instrument designed for surgical cardiac patients. Such instrument must take into account the severity of the congestive heart failure and the clinical signs of cachexia^[19]^. Several studies have shown the association between nutritional status and high incidence of complications in the postoperative period of major cardiac surgeries^[6,9,19,20]^.

The findings have shown high prevalence of clinical complications in the postoperative period of cardiac surgeries, with prevalence of non-infectious complications. Such data meet the findings by Soares et al.^[10]^, who found 58% prevalence of clinical complications. However, Lomivorotov et al.^[19]^ have observed this prevalence in 42% of the patients, only.

The renal complications were the most frequent ones among all the complications analyzed herein. They were followed by the hematological and infectious complications. These findings contradict another study, which showed that pulmonary complications were the most frequent ones. Such complications were followed by the cardiac and neurological ones^[10]^. These divergent results may be explained by the association between the postoperative complications and pre-existing diseases such as pulmonary diseases, as well as with smoking^[2]^, age^[21]^, impaired nutritional status^[19]^, and obesity^[20]^.

Age has been linked to the occurrence of postoperative complications^[21]^ and to postoperative mortality^[21,22]^, because the aging process leads physiological reserve losses. Thus, it affects different systems at different levels, as in the case of renal dysfunction^[9]^. Our findings did not show association between age and mortality, and between age and the presence of postoperative complications. However, the stratification showed association between age and renal dysfunction, and this result corroborates that of another study^[22]^.

Regarding the nutritional assessment, the BMI is used in clinical practices as nutritional status indicator, since it is a low cost and easy to measure method. The overweight values of most studied individuals are similar to those found in another study^[20]^, but they were higher than those found in other results^[6,9]^.

The association between BMI and mortality after cardiac surgeries remains controversial. Some studies found no significant correlation between high BMI levels and mortality^[9,20]^; however, other studies found positive association between high BMI and postoperative complications^[7,20]^.

The literature has shown that the BMI higher than 30 kg/m^2^ is predictive of increased risk of surgical site infections^[7]^. Contrary to such result, the current study found no association between BMI and the presence of clinical complications in the postoperative period of cardiac surgeries. Other authors showed findings similar to those in the current study^[6]^.

The bodily compartments and the adipose and muscle tissues showed no association with clinical complications. These findings meet the results found in valve heart disease patients subjected to cardiac surgery^[6]^.

The mean APM thickness values found in the current study differ from the results found by other authors^[6,23]^. The mean APM presented by valve heart disease patients subjected to valve replacement was lower than that found in the presented study. However, according to their study, 19% of the patients were classified as having some degree of malnutrition. Approximately 48% of these patients had significant lean body mass loss in the preoperative period^[6]^. These data contradict the findings in the present study, which has classified most of the patients as overweight.

Lameu et al.^[14]^ assessed 421 healthy subjects and found values similar to those found in the current study, mean 11.5±2.7 mm. It is worth highlighting that much of the studied population comprised overweight patients and some of the analyzed anthropometric indicators were related to body fat distribution; all indicators were proportional to the adiposity increase.

Bragagnolo et al.^[23]^ performed a cross-sectional study comprising 87 patients eligible for major surgery in order to determine the reliability of APM thickness and its correlation with other anthropometric, biochemical and clinical parameters. Their results showed that APM thickness was a reliable method to assess the nutritional status of patients undergoing surgery.

There was significant association between APM thickness in the non-dominant hand and infectious complications, although a study performed with patients subjected to valve replacement has found such an association in patients with significant tropism loss in the adductor muscle, only (less than 6.5 mm)^[6]^. However, the study by Bragagnolo et al.^[23]^ showed an average for the adductor pollicis muscle thickness to detect 13.4 and 13.1 mm malnutrition in the dominant and in the non-dominant hand, respectively.

The CPB is used in most patients subjected to cardiac surgeries; however, it has been shown that CPBs longer than 90 minutes are predictors of renal complication development^[24]^, since there is induction of systemic inflammatory condition^[2,24]^. The CCU time is approximately four times longer in these patients, despite the association with increased hospital mortality^[22]^.

The studied patients showed significant association between CPB time and the herein studied complications, besides the association with cardiovascular complications. Thus, the current study corroborates another study. The causes of renal complications are multifactorial and have been related to the type of surgery, to surgery duration and to CPB time^[2,22,24]^.

Our results corroborate the findings of other authors who showed that serum albumin was significantly associated with renal complications, since hypoalbuminemia is a renal failure predictor^[7]^.

Serum albumin, which is routinely used as nutritional status marker, is sometimes inconsistent with malnutrition detection^[5]^, since it is influenced by factors such as catabolism^[5]^, inflammatory activity of the disease, hospitalization, and liver and kidney diseases^[19]^, although it is described as postoperative mortality prognostic indicator^[5,7]^. The findings in the present study show that albumin was not associated with death. Another variable associated with CPB time was the presence of pulmonary complications^[2]^.

With respect to the biochemical assessment, no difference was found between patients who had or not postoperative complications. However, these results contradict the literature, which shows association between TLC and infectious complications after cardiac surgeries^[6]^.

The results found herein in the studied population showed mean CCU time similar to that found in another study^[2]^; longer times were described by other authors^[10]^. A retrospective multicenter study performed in Brazil and a retrospective cohort study with patients undergoing cardiac surgery found mean stay time 3.8 days and 4 days in CCU, respectively^[2,25]^, thus confirming our findings. Stay time longer than two days has been defined as prolonged^[19]^. However, in another study, the median post-surgical CCU durations were 5 days and the median hospital stay was 16 days, opposing the hospitalization time found in the present study^[26]^.

Contrary to the results found in patients subjected to cardiac surgery, the mean hospital stay was higher than that described in the literature^[6,25,26]^.

The mortality rates found in the current study were lower than those found in patients undergoing heart valve surgery^[6,20]^. This variation may be explained by the age and profile of the studied population.

The data presented in the current study demonstrated that the impaired nutritional status of the patients has affected the surgical outcome after cardiac surgeries.

## CONCLUSION

The preoperative nutritional status may be associated with postoperative complications in patients undergoing major elective cardiac surgeries. Albumin was associated with renal complications and the adductor pollicis muscle thickness was associated with infectious complications. Therefore, it becomes extremely important to study nutritional status indicators because they are effective surgical risk predictors in patients undergoing cardiac surgeries.

**Table:** 

Authors’ roles & responsibilities	
LBG	Analysis and/or data interpretation; conception and design study; manuscript redaction or critical review of its content; realization of operations and/or trials; statistical analysis; final manuscript approval
NMTJ	Analysis and/or data interpretation; conception and design study; manuscript redaction or critical review of its content; realization of operations and/or trials; statistical analysis; final manuscript approval
MBG	Analysis and/or data interpretation; conception and design study; manuscript redaction or critical review of its content; realization of operations and/or trials; statistical analysis; final manuscript approval
LCGD	Analysis and/or data interpretation; conception and design study; manuscript redaction or critical review of its content; realization of operations and/or trials; statistical analysis; final manuscript approval
TCBJD	Analysis and/or data interpretation; conception and design study; manuscript redaction or critical review of its content; realization of operations and/or trials; statistical analysis; final manuscript approval
